# Dysregulated Responsiveness of Circulating Dendritic Cells to Toll-Like Receptors in ANCA-Associated Vasculitis

**DOI:** 10.3389/fimmu.2017.00102

**Published:** 2017-02-09

**Authors:** Cécile Braudeau, Antoine Néel, Karine Amouriaux, Jérôme C. Martin, Marie Rimbert, Audrey Besançon, Stéphanie Giraudet, Caroline Terrien, Marine Aliaga, Nina Salabert-Le Guen, Caroline Hémont, Mohamed Hamidou, Régis Josien

**Affiliations:** ^1^CIMNA, Laboratoire d’Immunologie, CHU Nantes, Nantes, France; ^2^Institut de Transplantation-Urologie-Néphrologie (ITUN), CHU Nantes, Nantes, France; ^3^Centre de Recherche en Transplantation et Immunologie (UMR1064), INSERM, Université de Nantes, Nantes, France; ^4^Faculté de Médecine, Université de Nantes, Nantes, France; ^5^Service de Médecine Interne, CHU Nantes, Nantes, France; ^6^LabEx Immunotherapy Graft Oncology (IGO), Nantes, France

**Keywords:** ANCA-associated vasculitis, dendritic cells, toll-like receptor, IL-12/IL-23p40, dysregulation

## Abstract

**Objective:**

Dendritic cells (DCs) are critical effectors of innate and adaptive immunity playing crucial roles in autoimmune responses. We previously showed that blood DC numbers were reduced in autoimmune antineutrophil cytoplasmic autoantibody-associated vasculitis (AAV). Here, we assessed toll-like receptor (TLR) responsiveness of blood DCs from patients with granulomatosis with polyangiitis (GPA) or microscopic polyangiitis (MPA).

**Methods:**

Blood samples from healthy controls (HCs), GPA, or MPA patients, without treatment, during acute phase (AP) or remission phase (RP) were analyzed. Cytokine production by DCs and T cells was assessed on whole blood by flow cytometry after TLRs or polyclonal stimulation, respectively.

**Results:**

We first showed that GPA and MPA are associated with a decreased blood DC number during AP. Conventional DCs (cDCs) from patients with GPA and MPA in AP exhibited a profound decrease of IL-12/IL-23p40 production after TLR3, 4, or 7/8 stimulation compared to patients in remission and HC, with a return to normal values in RP. TNFα secretion was also affected, with a decrease in cDCs from GPA patients in AP after TLR3 stimulation but an increase after TLR7/8 stimulation. By contrast, the responsiveness of plasmacytoid DCs to TLR7 and 9 was only marginally affected. Finally, we observed that IFNγ-producing CD4^+^ T cell frequency was significantly lower in AP-GPA patients than in HC.

**Conclusion:**

We describe, for the first time, a dysregulated response to TLRs of circulating DCs in AAV patients mostly affecting cDCs that exhibit an unexpected reduced inflammatory cytokine secretion possibly contributing to an altered Th cell response.

## Introduction

Antineutrophil cytoplasmic autoantibody (ANCA)-associated vasculitis (AAV) are chronic and systemic autoimmune diseases characterized by small vessels inflammation and necrosis that can involve many organs and tissues (lungs, kidneys, heart, gut, nervous system, skin, etc.). Vascular inflammation, presence or absence of granulomatosis and/or asthma, and ANCA specificity currently help to define three disease entities, namely, granulomatosis with polyangiitis (GPA), microscopic polyangiitis (MPA), and eosinophilic granulomatosis with polyangiitis (EGPA) (1–3). The disease severity is evaluated and followed using the Birmingham Vasculitis Activity Score (BVAS) that takes into account the damage of the different organs affected by the disease (4). Classification and diagnostic of vasculitis are in fact complicated due to heterogeneous nature of these diseases, which comprise a wide range of clinical signs. ANCA are identified by indirect immunofluorescence on fixed neutrophils smears and are mandatory for the diagnosis. ANCA are mostly directed to two neutrophil granule proteins, proteinase 3 (PR3) and myeloperoxidase (MPO). Their specificity is important in clinical practice as PR3-ANCA and MPO-ANCA are strongly associated with GPA and MPA, respectively, whereas the association with EGPA is much less clear (3, 5, 6). Actually, recent studies led several authors to propose to primarily classify AAV based on ANCA specificity rather than clinical features (7–9). GWAS studies have shown a strongest association of genetic polymorphisms with ANCA specificity rather than with the clinical definition of GPA vs MPA (10, 11). Geographical disparities have also been found with PR3-AAV being more prevalent in northwestern Europe and North America, while MPO-AAV is more prevalent in southern Europe, Asia, and the Pacific, possibly due to genetic and environmental factors (8). In fact, this association also extends to some clinical (9) and biological (12, 13) parameters.

Pathogenic mechanisms in AAV are not clearly elucidated, but extensive evidence argues for a deleterious role of ANCA as effectors of tissue damage (5). ANCA induce vasculitis by activating circulating primed neutrophils and causing them to penetrate and damage vessel walls by undergoing respiratory burst, degranulation, NETosis, apoptosis, or necrosis. Moreover, ANCA-activated neutrophils also release factors that activate the complement and contribute to inflammation (14). Interestingly, it has been shown that whereas both MPO-ANCA and PR3-ANCA can bind and be internalized by endothelial cells, they actually exert different effects: PR3 inducing apoptosis, whereas MPO inducing production of intracellular reactive oxygen species (12). Mechanisms leading to autoimmune response induction and maintenance in AAV are, on the other hand, poorly understood but could be favored by decreased immunoregulatory mechanisms involving regulatory T and/or B cells (15–19). More recently, Millet et al. suggested another defect in immunoregulatory mechanisms in GPA by showing that the presence of PR3 on the membrane of apoptotic neutrophils impeded the immunosuppressive effect of apoptotic cell efferocytosis and promoted sustained inflammation (20). The presence of phosphatidylserine-associated PR3 on apoptotic cells generated a proinflammatory microenvironment, which facilitates the differentiation of Th2/Th9 and Th1 CD4^+^ T cells through the interaction between plasmacytoid dendritic cells (pDCs) and naive T cells (20). The potential role of pathogenic Th subsets in AAV has mostly been studied on GPA and is actually far from clear. Indeed, previous studies have shown an increased frequency of circulating Th1 (21–23), whereas others reported decreased Th1 cells in GPA (24). An increased frequency of Th2 cells in blood (24) and nasal tissues (25) was reported in GPA. Another report showed, by contrast, large numbers of IFNg^+^ but not IL-4^+^ cells in nasal tissues in GPA (26). More recently, CD4^+^ T cells from GPA patients were also found to exhibit a skewed distribution of Th9 (20). In addition, we recently reported an important decrease in circulating mucosal-associated invariant T (MAIT) cells in AAV patient that persisted during remission suggesting a role for these innate-like lymphocytes in AAV (27).

Dendritic cells (DCs) are key antigen-presenting cells to naïve T cells that play critical roles to initiate and control adaptive immune responses (28, 29). DCs are heterogeneous and comprise two major populations, conventional DCs (cDCs) and pDCs. DC subsets differ in terms of cytokine production, T cell stimulation, and *in vivo* localization (28–31). DCs are activated through sensing exogenous or endogenous ligands that bind pattern recognition receptors, such as toll-like receptors (TLRs), whose expression pattern also differs among DC subsets. Following TLR activation, DCs upregulate costimulatory molecules expression and produce inflammatory cytokines that play crucial roles in T cell polarization (32), cDCs being major producers of IL-12 and pDCs major producers of type 1 IFN (33, 34).

We previously reported that blood cDCs and pDCs were strongly decreased in AAV patients during flares. This might be related to increased apoptosis of DCs due to systemic inflammation as it was recently shown for pDCs in a mouse model (35), or their recruitment in tissues (17). Supporting this latter hypothesis, we observed an increased expression of the adhesion molecule CD62L on cDCs and even more pronounced on pDCs from AAV patients as compared to DC from healthy controls (HCs) or AAV in remission (17). Very few studies actually analyzed the presence of DCs in AAV lesions. One study showed that CD208^+^ and CD209^+^ cells (presumably cDCs) clustered with T cells in renal biopsies of AAV patients (36), and another one identified DC-LAMP-expressing cells in GPA-granuloma in nasal biopsies (37). A very recent report identified IFNα-producing pDCs in close proximity to macrophages and apoptotic neutrophils within lung granuloma lesions in GPA patients (20). Based on these findings, we hypothesized that circulating DCs could have a semi-activated state in AAV patients and be a source of inflammatory cytokines. We therefore analyzed TLR-induced cytokine production by blood DCs from AAV patients using a whole blood (WB) assay and observed that circulating cDCs from GPA and MPA actually displayed a mostly reduced IL-12/IL-23p40 production in response to several TLR ligands, whereas the production of type I IFN by pDCs was preserved overall. Given the central role of DC in polarizing T cells, we also assessed on the same blood samples T cell subsets frequencies and cytokine production and found a decreased frequency of IFNγ-producing CD4^+^ T cells.

## Materials and Methods

### Patients

Fourteen HCs and 39 age- and sex-matched patients with AAV, comprising 25 GPA and 14 MPA, were included in this study (Table 1). Acute phase (AP) of AAV was defined by a BVAS >3 and remission by a BVAS = 0 (Table 1), according to EULAR activity criteria (38). AP patients were treatment free at time of blood sampling. Patients with flares or in remission had no immunosuppressive drugs for more than 3 months (1 patient) or more than 6 months (19 patients). HCs comprised 14 donors recruited either by the local Blood Bank (EFS Pays de la Loire) or our institution, who were 31–84 years old. Venous blood samples were collected in EDTA and heparin tubes and processed for analysis within 4 h. The study was approved by our local ethical committee (Comité de Protection des Personnes Ouest IV—Nantes), and all patients and HCs provided written informed consent.

**Table 1:** **Patients’ clinical and biological features at inclusion**.

Characteristics	GPA (*N* = 25)		MPA (*N* = 14)		HC (*N* = 14)
	Acute	Remission	Acute	Remission	
*n*	15	10	9	5	14
Mean age (range)	58 (23–82)	59 (35–74)	67 (41–84)	65 (54–81)	49 (31–84)
M/F	8/7	4/6	5/4	2/3	7/7
ANCA anti MPO/PR3	4/11	0/10	9/0	5/0	ND
BVAS mean (range)	13 (3–23)	0	12 (6–19)	0	ND
CRP mean (range) (mg/l)	93 (3–267)	4 (1–10)	86 (20–200)	7 (0–28)	ND
Kidney injury	8/15	4/10	5/9	3/5	ND
Creatinine level median (range) (μM)	83 (41–444)	70 (47–140)	195.5 (28–420)	96 (65–788)	ND
Last treatment before stop	AZA: 2	AZA: 5	AZA: 0	AZA: 3	
	MMF: 1	MMF: 1	MMF: 0	MMF: 0	
	MTX: 2	MTX: 1	MTX: 0	MTX: 0	
	RTX: 0	RTX: 3	RTX: 0	RTX: 0	
	Cs: 0	Cs: 0	Cs: 1	Cs: 1	
	CYC: 0	CYC: 0	CYC: 1	CYC: 0	
	None: 10	None: 0	None: 7	None: 1	
Duration of the disease at inclusion (range) (months)	42.4 (0–141)	93.1 (50–188)	35.6 (0–135)	64 (0–67)	
Relapse/presentation	5/10		2/9		

*ND, not determined; AZA, azathioprine; MMF, mycophenolate mofetil; MTX, methotrexate; RTX, rituximab; Cs, corticosteroids; CYC, cyclophosphamide; GPA, granulomatosis with polyangiitis; MPA, microscopic polyangiitis; HC, healthy control; ANCA, antineutrophil cytoplasmic autoantibody; BVAS, Birmingham Vasculitis Activity Score*.

### WB *In Vitro* Stimulation Assays

#### Dendritic Cells

Within a maximum of 4 h after drawing, 100 µl of heparinized WB samples were incubated 4 h with the following TLR ligands: heat-killed *Listeria monocytogenes* (HKLM, TLR2-L, 10^8^ HKLM/ml), Poly(I:C) (TLR3-L, 100 µg/ml), CL097 (imidazoquinoline compound, TLR7/8-L, 2 µg/ml) and CPG ODN2395 [Type C CPG oligonucleotide, TLR9-L, 50 µM, all obtained from Invivogen (Toulouse, France)], or lipopolysaccharides (from *Escherichia coli* O26:B6, TLR4-L, 0.1 µg/ml) purchased from Sigma-Aldrich (St. Louis, MI, USA). GolgiPlug (BD Biosciences, Le Pont de Claix) was added during the last 3 h of incubation to inhibit cellular cytokine release. Incubation in medium alone served as a negative control condition.

#### T Cells

Within maximum of 4 h after drawing, 50-µl heparinized WB samples were incubated with PMA (phorbol 12-myristate 13-acetate) and ionomycin both purchased from Sigma-Aldrich (St. Louis, MI, USA) and at 20 ng/ml and 1 µg/ml, respectively. GolgiStop (BD Biosciences) was added during the last 3 h of incubation to inhibit cellular cytokine release. Incubation in medium alone served as a negative control condition.

### Flow Cytometry

#### Dendritic Cells

Dendritic cells were characterized using the six-color flow cytometry assay as we described previously (17). Briefly, 100 µl of WB were incubated with the following antibodies: CD45-V500, lineage cocktail1-FITC, HLA-DR-APC-Cy7, CD123-PECy5, all from BD Biosciences and CD11c-PECy7 (Beckman Coulter, Marseille, France). Absolute numbers of DCs were determined using BD Trucount™ Tubes (BD Biosciences). Samples were analyzed using a BD FacsCanto II analyzer with DIVA software (BD Biosciences, Le Pont de Claix, France). The whole tube was acquired to ensure a minimum of 1,000 events in the Lin^−^ HLA-DR^+^ (total DCs) gate for each sample.

#### T Cell Subsets

Absolute counts of CD4 and CD8 T cells were determined with BD Multitest™ CD3/CD8/CD45/CD4 in BD Trucount™ Tubes. Naïve/memory T cell subsets were identified on WB using CD45-V500, CD3-V421, CD4-PECy5.5, CD8-APC-H7, CD45RA-PECy7, CCR7-PE (BD Biosciences), and with CXCR3-FITC, CCR6-APC (Ozyme, St. Quentin-en-Yvelines, France).

#### Intracellular Cytokine Staining in DCs

Following WB stimulation with TLR ligands, surface staining was first performed with CD45-V500, Lineage cocktail1-FITC, HLA-DR-APC-Cy7, CD123-PECy5, and CD11c-PECy7 mAbs. Samples were then lysed, fixed, and permeabilized with Cytofix/Cytoperm Plus (BD Biosciences) and stained with the following anti-cytokine mAbs TNFα-APC (BD Biosciences), IL-12/IL-23p40-eFluor450 (eBiosciences, Paris, France), and IFNα-PE (MiltenyiBiotec, Paris, France). Samples were analyzed by flow cytometry, and the frequencies of cytokine-producing cDC and pDC were assessed. The whole tube was acquired to ensure a minimum of 1,000 events in the Lin^−^ HLA-DR^+^ (total DCs) gate for each sample.

#### Intracellular Cytokine Staining in T Cells

For cytokine production analysis by T cell subsets, surface staining was first performed with CD45-PERCP, CD3-FITC, and CD8-APC-H7 antibodies (BD Biosciences). Samples were then lysed, fixed, permeabilized, and then stained with IL-17A-eFluor660 (eBiosciences), IFNγ-V450, or IL-5-APC or IL-21-PE (BD Biosciences) antibodies. Samples were analyzed by flow cytometry.

### Statistics

Statistical analyses were performed using GraphPad prism 5.0 software (GraphPad Software, San Diego, CA, USA). Mean comparisons were performed with the non-parametric Kruskal–Wallis test and Dunn’s multiple test for *post hoc* analysis. Differences were defined as statistically significant when *p < 0.05.

## Results

### Decreased Number of DCs in Both GPA and MPA

**Figure 1:**
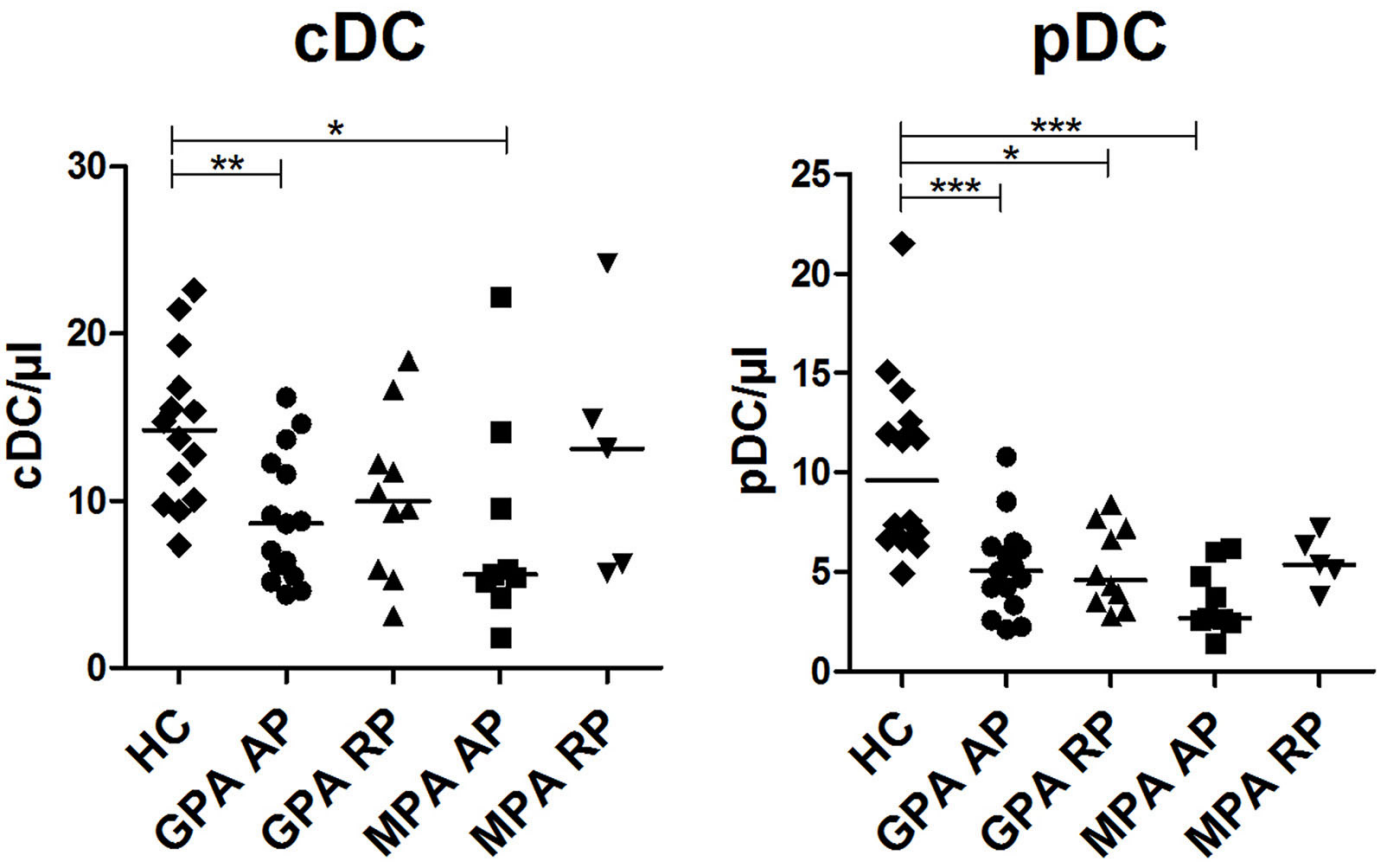
**Decreased number of dendritic cells (DCs) in granulomatosis with polyangiitis (GPA) and microscopic polyangiitis (MPA)**. Conventional DCs (cDCs) and plasmacytoid DCs (pDCs) were enumerated by flow cytometry on whole blood samples from patients with antineutrophil cytoplasmic autoantibody-associated vasculitis and healthy control [HC: *n* = 14, GPA-acute phase (AP): *n* = 15, GPA-remission phase (RP): *n* = 10, MPA-AP: *n* = 9, MPA-RP: *n* = 5].

We previously showed that patients with AAV displayed decreased numbers of circulating cDCs and pDCs during flares and to a lesser extent in remission phase (RP) (17). Here, we aimed to reproduce these data in a new cohort and assess whether differences could exist between GPA and MPA. Absolute counts of cDCs were significantly decreased in patients with GPA and MPA in AP compared to HCs (Figure 1, **p < 0.01, *p < 0.05, respectively). There was a trend to return to normal values in RP. A strong decrease of pDC absolute counts was also found in patients with active GPA and MPA (Figure 1, ***p < 0.001), as well as in RP GPA (*p < 0.05), but not in RP MPA despite a trend toward lower counts.

### Altered Function of cDCs during AAV Flares

**Figure 2:**
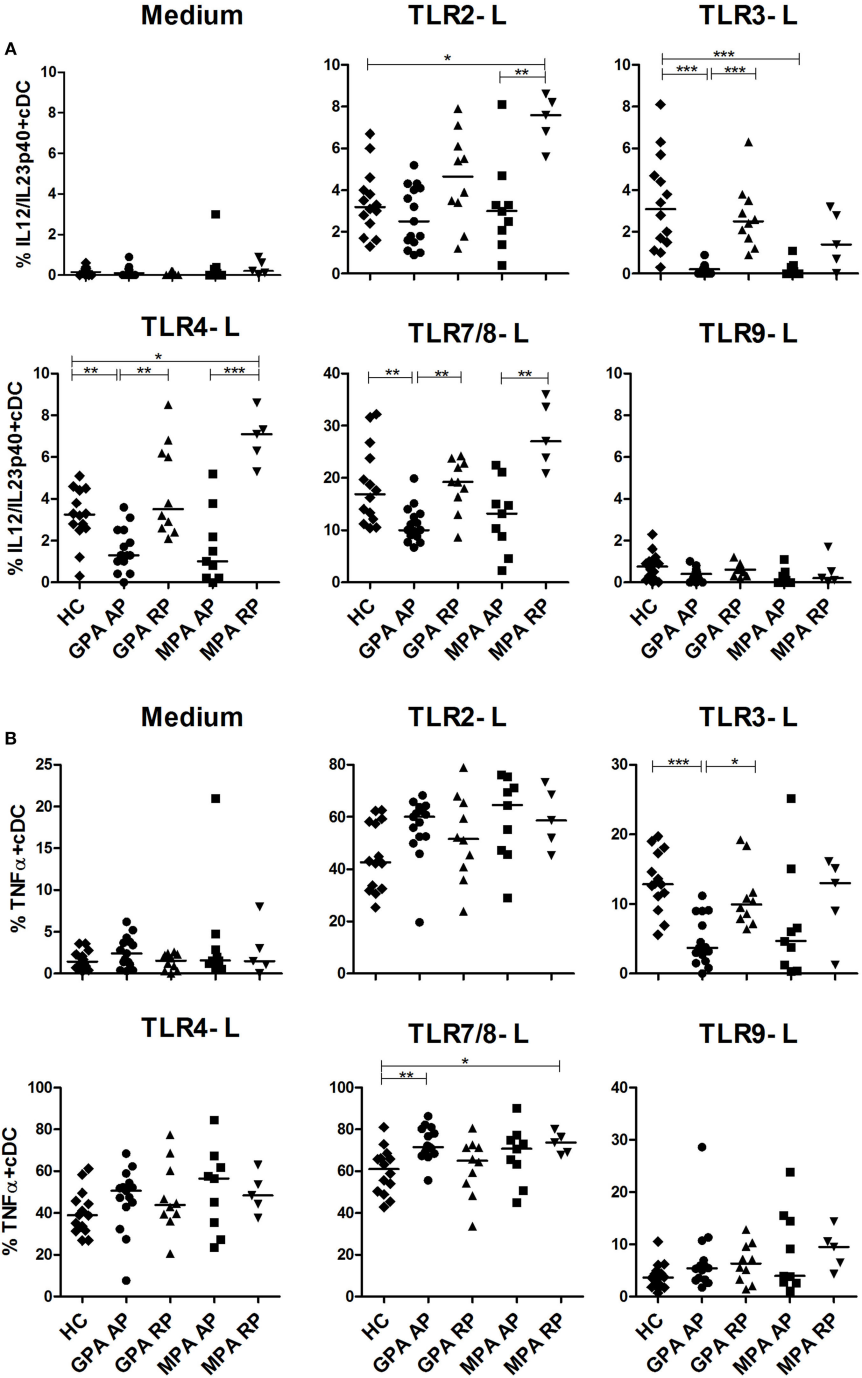
**Altered production of IL-12/IL-23-p40 and TNFα in stimulated conventional dendritic cells (cCDs)**. Whole blood samples were incubated with medium or toll-like receptor (TLR) 2, 3, 4, 7/8, or 9 ligands for 3.5 h and then stained for identification of cDCs (HLA-DR^+^, Lin^−^, CD11c^+^, CD123^−^) together with intracellular cytokine production IL-12/IL-23p40 **(A)** and TNFα **(B)**. Healthy control (HC): *n* = 14, granulomatosis with polyangiitis (GPA)-acute phase (AP): *n* = 15, GPA-remission phase (RP): *n* = 10, microscopic polyangiitis (MPA)-AP: *n* = 9, MPA-RP: *n* = 5.

We then assessed intracellular cytokine production of blood DCs by flow cytometry after stimulation by several TLR ligands with previous detailed method (39). We first investigated the production of the p40 chain which is shared by IL-12 and IL-23 (Figure 2A), two cytokines playing a critical role in inflammation and Th1 and Th17 CD4^+^ T cell polarization, respectively. In AP GPA, we observed a strong decrease of IL-12/IL-23p40 production by cDCs in response to TLR3-L (***p < 0.001) and more modestly to TLR4-L (**p < 0.01) and TLR7/8-L (**p < 0.01) as compared to HC, whereas no difference was detected for RP GPA. A similar pattern was observed in MPA, but significance was only reached with TLR3 stimulation (***p < 0.001). By contrast, cDCs from MPA in remission, but not GPA, exhibited an increase production of IL-12p40 upon TLR2 and TLR4 stimulation as compared to HC. As expected, TLR9 ligand did not induce IL-12 production in cDCs.

Concordant with a general alteration cDC responsiveness in AP GPA patients, TNFα production was also decreased after TLR3 stimulation (Figure 2B) (HC: ***p < 0.001), with a return to a normal level in RP (*p < 0.05). The same trend was observed for MPA patients without reaching significance. By contrast, after stimulation with TLR7/8 ligand, cDCs from active GPA and RP MPA patients exhibited increased production of TNFα compared to HC (**p < 0.01, *p < 0.05). However, the production of TNFα after TLR2, 4, and 9 stimulations was not altered.

### Production of IFNα by pDCs Is Preserved during Active AAV

**Figure 3:**
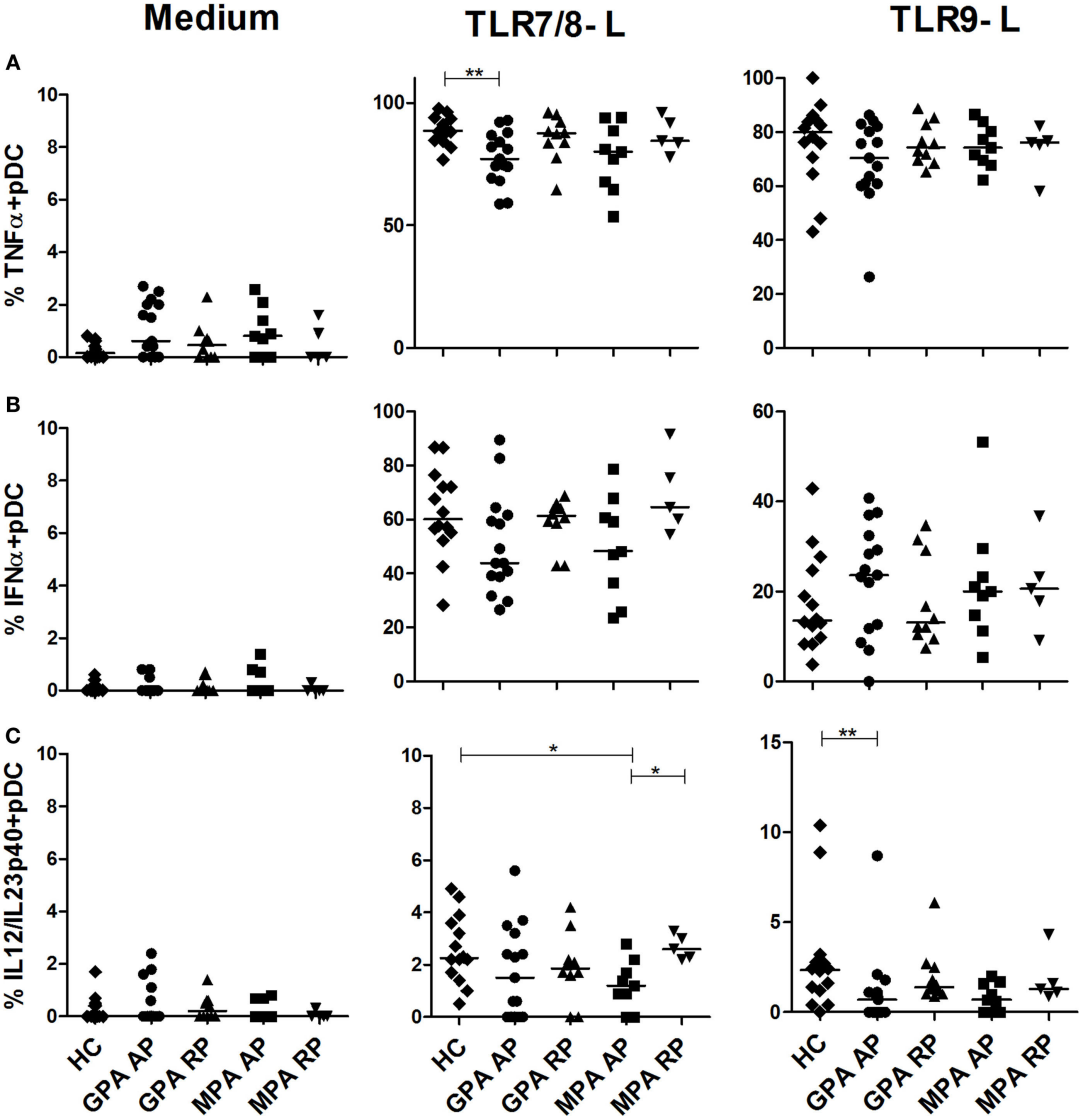
**Slight decrease of cytokine production by plasmacytoid dendritic cells (pDCs) in active antineutrophil cytoplasmic autoantibody-associated vasculitis**. Whole blood samples were incubated with medium or toll-like receptor (TLR) 7/8 or 9 ligands for 3.5 h and then stained for identification of pDCs (HLA-DR^+^, lin^−^, CD11c^−^, CD123^+^) together with intracellular cytokine production TNFα **(A)**, IFNα **(B)**, and IL-12/IL-23p40 **(C)**. Healthy control (HC): *n* = 14, granulomatosis with polyangiitis (GPA)-acute phase (AP): *n* = 15, GPA remission phase (RP): *n* = 10, microscopic polyangiitis (MPA)-AP: *n* = 9, MPA-RP: *n* = 5.

We also investigated if pDC responsiveness from AAV patients was altered after TLR7/8 or TLR9 stimulation (Figure 3). A slight, but significant, reduction of TNFα production was observed in pDCs from AP GPA patients after stimulation with the TLR7/8 ligand (Figure 3A, **p < 0.01). We observed that pDCs from AAV patients displayed a normal response after TLR9 stimulation in both phases of diseases and a normal IFNα production (Figure 3B). Low production of IL-12/IL-23p40 was observed in pDCs from HC that was further decreased in AP-MPA patients in response to TLR7/8 ligand (*p < 0.05) and in AP-GPA after TLR9 stimulation (**p < 0.01) (Figure 3C).

### Impaired IFNγ Production in CD4^+^ T Cells during AP of GPA

**Figure 4:**
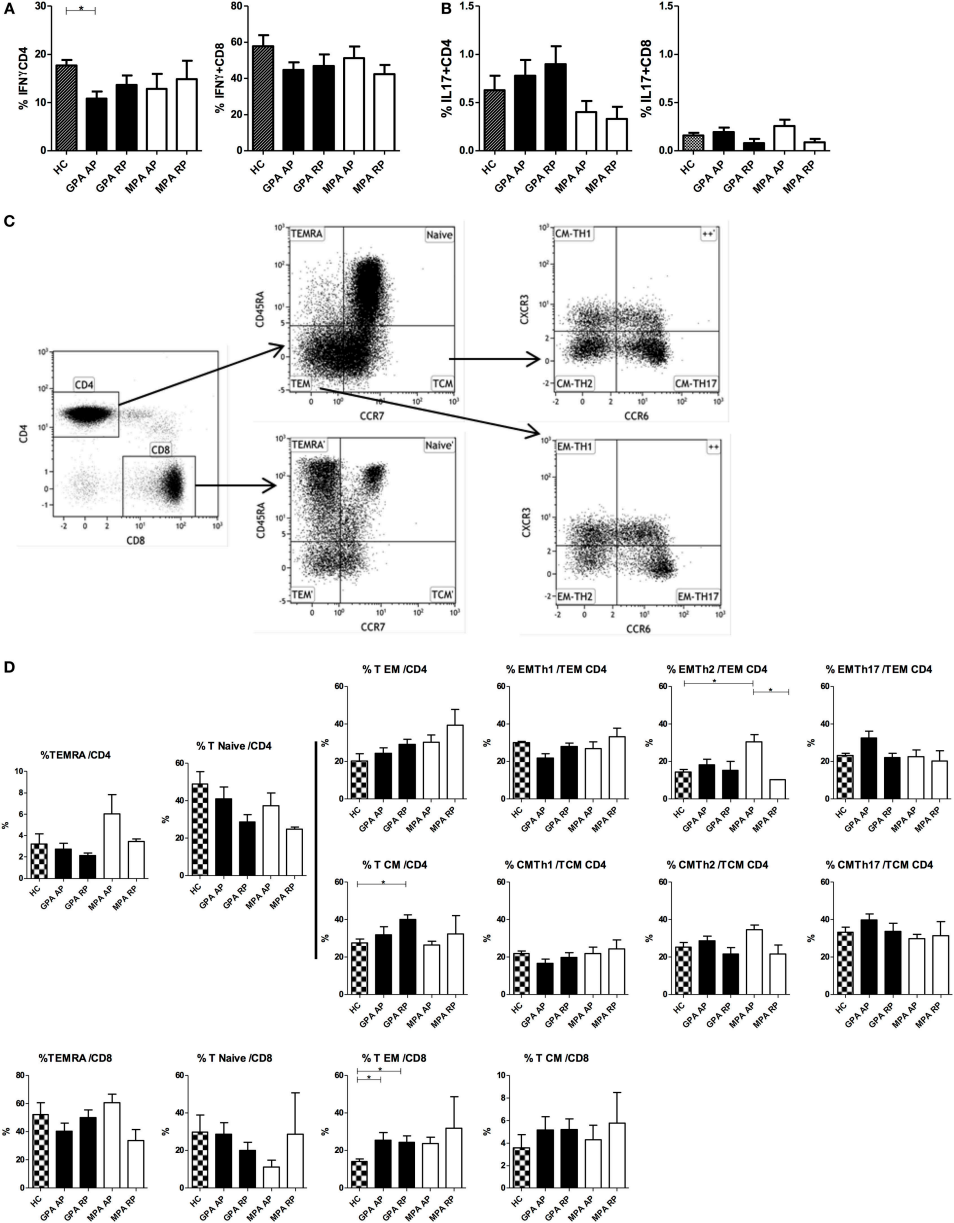
**Cytokine production and T cell subsets in antineutrophil cytoplasmic autoantibody-associated vasculitis**. Intracellular production of IFNγ **(A)** and IL-17 **(B)** was assessed by flow cytometry in whole blood (WB) in CD4^+^ and CD8^+^ T cells after PMA and ionomycin stimulation in healthy control (HC): *n* = 10, granulomatosis with polyangiitis (GPA)-acute phase (AP): *n* = 15, GPA-remission phase (RP): *n* = 10, microscopic polyangiitis (MPA)-AP: *n* = 9, MPA-RP: *n* = 5. Frequencies of T cell subsets in WB using flow cytometry **(C,D)**. Gating strategy to identify naïve/memory T cell and Th subsets **(C)**. T cell subsets in HC: *n* = 7, GPA-AP: *n* = 9, GPA-RP: *n* = 9, MPA-AP: *n* = 7, MPA-RP: *n* = 2 **(D)**. Results are expressed as percentage of positive cells.

Considering the decreased induction of IL-12/IL-23p40 in cDCs during AP, we hypothesized this could reverberate on Th1/Th2/Th17 responses, and we analyzed cytokine production by T cells after polyclonal stimulation as well as % of T cell subsets in our cohort (Figure 4). Interestingly, frequencies of IFNγ-producing CD4^+^, but not CD8^+^, T cells were significantly reduced in AP GPA, but not MPA, as compared to HC (*p < 0.05, Figure 4A). A tendency to increased frequencies of IL-17-producing CD4^+^ T cells was also observed in GPA only but without reaching statistical significance (Figure 4B). No perturbation in IL-5 and IL-21 production by CD4^+^ and CD8^+^ T cells was observed during AAV (data not shown).

Frequencies of Th subsets were also assessed using CXCR3 and CCR6 expression that are useful surface markers for functionally distinct memory T cell subsets: Th17 (CXCR3^−^CCR6^+^), Th2 (CXCR3^−^CCR6^−^), Th1/Th17 (CXCR3^+^CCR6^+^), and Th1 (CXCR3^+^CCR6^−^) (40, 41). The gating strategy is depicted in Figure 4C. No difference was found in naïve (CD45RA^+^CCR7^+^) and TEMRA (CD45RA^+^CCR7^−^) CD4^+^ and CD8^+^ T cells between all groups (Figure 4D). In CD4^+^ effector memory subsets, we found a significant increase of CXCR3^−^CCR6^−^ (Th2) T cells in AP MPA (*p < 0.05). Central memory CD4^+^ T cells were increased in RP GPA but with no modification of Th1, Th2, and Th17 subsets frequencies. In the CD8 compartment, we observed an increase frequency of effector memory T cells (CD45RA^−^CCR7^−^) in GPA patients as compared to HC (*p < 0.05) and a similar trend in MPA, suggestive of increased CD8^+^ T cell activation in AAV.

## Discussion

Although ANCA play a central role in vascular lesions and inflammation during AAV, the potential role of DCs in regulating autoimmune adaptive responses in these diseases is poorly understood. We first extended our previous findings of a decrease number of blood DC subsets during AAV (17) by showing that this holds true in both GPA and MPA. Given that DCs are major producers of cytokines regulating T-cell activation, we sought to determine whether the capacity of blood DCs to produce cytokines in response to TLR ligands was increased in AAV. We in fact mostly observed a decreased responsiveness of blood cDCs from AAV patients to various TLR ligands. The WB assay we used in this study presents with several advantages. First, it avoids non-specific activation of DCs that is usually associated with isolation procedures. Second, it requires only small volume of blood (2 ml). Third, we previously showed using this assay that pDCs responded only to TLR7/8 and TLR9 ligands by producing mainly IFNα and TNFα, but not IL-12p40, whereas cDCs responded to all TLR ligands, but not TLR9, by producing IL-12p40 and TNFα, but not IFNα (39, 42). These results, which are similar to what has been shown with purified blood cDCs and pDCs with respect to their pattern of response to TLR and frequencies of cytokine-secreting cells (43), led us to consider that this assay mostly assesses the direct effect of TLR ligands on DC subsets. One limit of our assay is however that it assessed cDC and pDC responses to single TLR stimulation, and it is likely that these experimental conditions do not measure the maximum capacity of each DC subset to produce cytokines. Indeed, it is well known that optimal secretion of IL-12p70 by human cDCs requires combination of several signals such as a TLR + CD40L (44), TLR + inflammatory cytokines (45), or several TLRs (46).

Circulating DCs have been analyzed in various autoimmune diseases, and their numbers have mostly been reported to be reduced (47). As the presence of both mDCs and pDCs have been demonstrated in inflammatory tissues such as skin, synovial fluids, or muscle for instance, the reduction in circulating DC numbers has been proposed to reflect their increased recruitment in these tissues. For instance, in lupus, pDCs are reduced and activated in the blood and are found in large number in some inflammatory tissues (48, 49). By contrast, this is much less clear for mDCs whose migration to inflammatory tissues has not been documented and that are in fact heterogeneous (50). To our knowledge, the responsiveness of circulating mDCs to TLR has not been assessed previously in autoimmune diseases. Our data show that patients with AAV exhibit a profound and rather complex dysregulation of TLR responsiveness. During the AP of AAV, we observed a significant defect of IL-12/IL-23p40 production by cDCs after stimulation with several TLR ligands (TLR3, 4, and 7/8), with an apparent return to basal value during remission. This contrasts with previous studies that have shown an increased production of IL-12p70 by activated blood monocytes isolated from active GPA patients as compared to HD (23, 51), which was furthermore normalized by therapy (51). TNFα secretion by cDCs was also affected in AAV, but only after stimulation with TLR 3 or 7/8 ligands. Intriguingly, during active GPA, the frequencies of TNFα-producing cDCs after TLR3 stimulation were decreased but were increased after stimulation with TLR7/8 ligand. As cDCs comprise two subsets, namely, CD1c^+^ cDCs and CD141^+^ cDCs (42), it is possible that this dysregulation in TLR responsiveness and cytokine production reflect changes in these cDC subset frequencies. However, our unpublished and preliminary data indicate that both CD1c^+^ and CD141^+^ cDCs are decreased during AAV flares. These extremely low frequencies of CD141^+^ cDCs in AAV patients precluded the possibility of assessing their cytokine production using the WB assay. In contrast to cDCs, pDC functions were only slightly affected with no modification in the main cytokine produced by pDCs, i.e., IFNα. Taken together, these results point to a peculiar defect in IL-12p40 production in cDCs during AAV. It remains to be determined whether this will translate in reduced IL-12, IL-23, or both cytokine activities. It will obviously be important to assess the other functions of DCs in AAV patients such as antigen presentation and T cell stimulation activity. However, this is technically challenging due to the frequencies of these cells.

The molecular mechanism for this altered TLR-induced cytokine secretion by cDCs in AAV, especially during the AP, still remains to be understood. One possibility is that this is related to a modulation of TLR expression in DCs, but isolating blood DCs for qPCR experiments would require large volumes of blood and was not technically feasible in this study. As mAb to several TLRs are now available, it would be interesting to assess TLR expression by FACS on cDCs in future studies. Of note, Tadema et al. reported an increased expression of TLR2 and TLR4 on monocytes in AAV patients but no difference in TLR expression between patients in AP and RP (52). By contrast, Holle et al. reported no difference in the expression and activation of TLR2, TLR4, and TLR9 on PMNs in GPA compared to HCs (53). Many single-nucleotide polymorphisms (SNPs) in genes that encode TLRs and their signaling molecules have been associated with human disease progression and susceptibility (54). Although Husmann et al. recently reported on the association between four SNPs in TLR9 gene, we observed that the response of pDC to TLR9 in our assay was normal (55). Another possibility is that intracellular TLR-mediated signaling is altered in DCs from AAV patients. However, the defect we observed involved both MyD88-dependent (TLR7/8) and MyD88-independent (TLR3) TLRs, suggesting that proximal signalization is not involved. The fact that a decreased IL-12p40 secretion was observed after TLR3, 4, or 7/8 triggering could suggest a defect in distal NF-κB-dependent gene regulation in cDCs during AAV (56). Importantly, we only included untreated AAV patients during AP in our study excluding that the observed altered DC functions could be related to immunosuppressive drugs such as corticosteroids that are well known to inhibit the NF-κB pathway. The role of a soluble factor specific to AAV that would inhibit TLR responsiveness of cDCs, but not pDCs, can not be excluded. In fact, a recent report showed that serum from active AAV patients promoted polarization toward the M2c subtype macrophages, expressing low levels of IL-12 and TNFα and increasing phagocytosis capacity (57).

Using the same assay, we recently reported a reduction in cDC numbers but without IL-12p40 dysregulation in untreated patients suffering from Gaucher disease (39), a form of genetic lysosomal storage disease associated with a strong systemic inflammatory response. Therefore, this suggests that the defect in TLR response we observed in AAV patients is not simply related to systemic inflammation. The numbers of circulating mDCs and pDCs were also shown by others to be reduced in the blood of rheumatoid arthritis patients and to inversely correlate with serum CRP levels (58). Although the numbers of mDCs and pDCs did not correlate with CRP in our AAV patients, it did do with BVAS (data not shown), suggesting again that this decrease is not merely a reflection of systemic inflammation. It would however be important to investigate whether the altered innate function of cDCs we observed in AAV is also observed in other autoimmune inflammatory diseases. An important limit of our study is that we only assess the responsiveness of blood DCs and whether this could reflect the function of DCs infiltrating inflammatory tissues is unknown. In spite of an important reduction in circulating pDCs in active AAV, the innate function of these cells appeared well preserved. Supporting this statement, a recent report in GPA patients showed that pDCs are present in inflammatory issues such as the lungs in which they appear to secrete IFNα (20). Interestingly, using a murine model, the same group demonstrated to role for pDCs in driving pathogenic T cell responses (20). Regarding cDCs, their presence have been reported in renal (36) and nasal (25) lesions during AAV but whether their capacity to produce cytokines is altered has not been assessed. Investigating the function of these tissues cDCs would therefore be important to understand the significance of our observation in blood DCs. One can hypothesize that this dysregulation of DC responsiveness to TLR is important to reduce inflammatory response. However, by limiting the response to TLR engagement during AP, this could possibly impair immune responses to pathogens in AAV patients and play a role in the reported susceptibility of these patients to some infections (59, 60).

The main function of DCs is to stimulate naïve T cells and drive their differentiation. Interestingly, we observed a reduction in the frequencies of IFNγ-producing CD4^+^ T cells in the blood of AP GPA as compared to HC, confirming a previous report (24). It is tempting to relate this to the defect of IL-12/IL-23p40 observed in DCs, but further investigations are required to demonstrate this link. A tendency to increased frequencies of IL-17-producing CD4^+^ T cells was observed in GPA, as already found in several studies (20, 61, 62). Although the potential role of Th17 cells in AAV remains to be demonstrated, recent data indicate that Th17 cells are important effector cells in humoral autoimmune diseases (63). T cell subset analysis showed also discrete modifications regarding the proportion of naïve vs memory CD4^+^ T cells that actually confirm previous studies showing a persistent expansion of CD4^+^ effector memory T cells, with a reciprocal decrease in naïve CD4^+^ T cells in AAV patients (64, 65). Accordingly, infiltrating T cells in lung lesions and glomeruli were shown to consist mainly of CD4^+^ T cells with a memory phenotype (66, 67). However, an important limit of our study is that we analyzed polyclonal and not antigen-specific responses of T cells.

Our study also indicates that GPA and MPA exhibit some subtle difference regarding the function of DC and the frequencies of T cells. Both GPA and MPA are associated with an important decrease in circulating cDCs and pDCs during flares and very similar dysregulation of TLR responsiveness with a decrease response to TLR3, 4, and 7/8. However, during remission, DCs from MPA, but not GPA, patients exhibited an increased IL-12p40 production upon TLR2 and TLR4 stimulation suggesting a hyperactivated state. As TLR2 and 4 are mostly involved in the recognition of Gram+ and Gram− bacterial wall, it will be interesting to address the role of this apparent increase DC responsiveness in the absence of role of *Staphylococcus aureus* carriage for MPA relapses (68, 69). Another difference was the increase frequency of “Th2” effector memory cells in MPA vs HC and GPA during AP. It should be noted that classifying our patients according to their ANCA specificity rather than to the clinical syndrome did not change the results of our study as all MPA patients had MPO-ANCA and only 5 out of 25 GPA patients had MPO-ANCA rather than PR3-ANCA.

In summary, we describe for the first time, a dysregulated response to TLRs of blood DCs in AAV patients as evidenced by altered cytokine production, with previously unreported defect of IL-12/IL-23p40 pathway. Together with our recent report on the persistent decrease of MAIT cells in the same patients (27), these results point to a role for altered innate immune responses in the pathogenesis of AAV. How this impacts on the auto-antigen-specific and pathogenic immune response remains to be determined.

## Ethics Statement

This study was carried out in accordance with the recommendations of our Institutional Review Board (CPP OUEST IV) with written informed consent from all subjects. All the subjects gave written informed consent in accordance with the Declaration of Helsinki.

## Author Contributions

CB and AN participated in the design of the study, carried out the experiments, and prepared the manuscript. MH, MR, and JM participated to the conception of the study, contributed to the discussion, and reviewed the manuscript. KA, AB, SG, CT, MA, NS, and CH performed experiments. RJ designed the study, supervised the project, and wrote the final manuscript.

## Conflict of Interest Statement

The authors declare that the research was conducted in the absence of any commercial or financial relationships that could be construed as a potential conflict of interest.
